# Supplementary material for: Ergonomic assessment of work-related musculoskeletal disorder and its determinants among commercial mini bus drivers and driver assistants (mini bus conductors) in Nigeria
Source: PLoS One. 2021 Dec 7;16(12):e0260211. doi: 10.1371/journal.pone.0260211 (PMC8651118; doi:10.1371/journal.pone.0260211)
Supplement: Attachment — Submitted filename: Response to Reviewers.docx [file pone.0260211.s001.docx]

**July 5th, 2021**

**Editor, PLOS One**

Dear Editor:

Thank you for your interest in our manuscript (PONE-D-21-05678) titled, *"Ergonomic Assessment of Work-Related Musculoskeletal Disorder and its Determinants among Commercial Mini Bus Drivers and Driver Assistants (Mini Bus Conductors) in Nigeria".* We have carefully read your comments and recommendations and find them to be helpful. We have taken great care to present our commentary with accuracy, precision, and clarity.

To facilitate your evaluation of our revised manuscript, we have incorporated all the reviewers’ comments (*italicized*) in our reply **(bold)** and addressed the requests with specific point-by-point responses to each query. We separately delineated and numbered each query with a corresponding response. All editions, additions and changes in the manuscript were done using the MS Word Track change Highlight**.** We also indicated the precise location in the revised manuscript where we have addressed your comments. All references to the location of our changes (i.e., page) correspond to the revised manuscript.

**Editor:**

*1) We note that Figures 1 to 3 includes an image of a participant in the study.
As per the PLOS ONE policy (http://journals.plos.org/plosone/s/submission-guidelines#loc-human-subjects-research) on papers that include identifying, or potentially identifying, information, the individual(s) or parent(s)/guardian(s) must be informed of the terms of the PLOS open-access (CC-BY) license and provide specific permission for publication of these details under the terms of this license. Please download the Consent Form for Publication in a PLOS Journal (http://journals.plos.org/plosone/s/file?id=8ce6/plos-consent-form-english.pdf). The signed consent form should not be submitted with the manuscript, but should be securely filed in the individual's case notes. Please amend the methods section and ethics statement of the manuscript to explicitly state that the patient/participant has provided consent for publication: “The individual in this manuscript has given written informed consent (as outlined in PLOS consent form) to publish these case details”.*

**Response:**

**The statement “The individual in this manuscript has given written informed consent (as outlined in PLOS consent form) to publish these case details including the photograph” has been explicitly stated in the Method section (Ethics Statement) as seen on page 5, lines 25 – 27.**

**Reviewer #1:**
*This article evaluates work-related musculoskeletal disorder and its determinants among Nigerian commercial bus driver and bus conductors. The topic is important, however, the presentation and writing of the article needs to be substantially improved. Also, some key information and results should be explained in detail.*

**Response: Thank you for your review and finding the topic important. We have made effort to improve the presentation and writing of the article. Details of key information and results have been equally explained in detail.**

i. *The article lacks a clearly formulated research question and hypotheses. The choice of measures and the report of the results is therefore difficult to follow and seems arbitrary.*
   **Response: Attempts have been made to clearly formulate the research question and hypotheses that will hopefully make the choice of measure and report of the result easier to follow and understand (See page 3)**
ii. *The literature review is sparse. Could the authors include more literature on biomechanic assessment that could be used to give precise recommendations as to how the job or the spatial constraints inside a bus would need to change to improve the working situation of the drivers and conductors?*
    **Response: Further literature review on the biomechanics of prolonged sitting and standing and how they relate to MSD pathology has been done (See page 2)**
iii. *More information about the procedure would be appreciated. How exactly where the interviews conducted?*
     **Response: This study had a quantitative design, there was no qualitative interviews. Also, the instruments used in this study were client-administered. Only minimal guidance in understanding how to fill these instruments were given. This information has also been provided (See page 6)**
iv. *The authors claim that there is an urgent need for ergonomic training and intervention for both bus drivers and conductors tailored to address this public health challenge. Is this a feasible countermeasure or does the overall occupational, legal situation need to change? Could the authors derive more specific guidelines on what needs to change?*
    **Response: Thank you for this very important suggestion. Changing the overall occupational and legal situation is a possible approach but may take a much longer time to be implemented. More so, the targeted persons may not understand the merits behind it and may result in the problem of compliance. However, education should be the first line of action before or alongside this important suggestion. This has been added to the discussion (See page 14)**
v. *Formatting: The article lacks the necessary line numbers. This makes it difficult to provide detailed annotations. The citation style does not meet PLOS requirements and is partially misplaced.*
   **Response: Sorry about the difficulty. This has been done.**
vi. *The relevant data are not within the manuscript and its supporting Information files.*

**Response: Pardon me, I am not sure I understood what you meant by the above statement. Kindly let me know the particular missing relevant data and supporting information**

vii. *The authors should elaborate more on the rationale for their measures. E.g., why was a pulse rate measured and reported? Pulse rate is a very instantaneous measure.*

**Response: Some physiological variables such as pulse rate, blood pressure etc are health indices and so can be relied upon to make inference on the health status of the participants. These variables sometimes indicate the level of mental stress the person is exposed to. These are relevant vital signs for health.**

**viii.** *Prediction Model: Is it reasonable to predict the probability of receiving MSDs for participants who already show such symptoms. Could the authors elaborate on why they chose this statistical method? Should the dataset be divided into participants who already show MSD and those who do not?*
**Response: The prediction model was used to identify the important ergonomic determinants of WMSD in these cohorts. This result can be relied upon subsequently in preventing further occurrence or its worsening. The identified predictors can also be used in risk/hazard analysis and for education as a preventive measure.**

**Dividing the dataset into those with MSD and those without would imply a case-control design and will require a different analysis altogether even though this is not our design and objective.**

**ix.** *Figure 1 is blurred and not properly selected to represent the authors’ intent (e.g., the back support is barely visible). Why is it important that the driver's neck is flexed in this figure? How is this consistent with the figure description in the text?*

**Response: Thanks for your observation. This was a still picture taken from a driver who doubled as a conductor. He was making efforts to communicate with some passengers and this forced him into some awkward posture. This practice is not uncommon in the study environment and so the picture attempts to further elucidate on the research problem (see page 2, line 13)**

**Reviewer #2:**

*This paper presents study that assess the determinants of musculoskeletal disorders among commercial bus drivers and bus conductors in Nigeria with consideration of an ergonomics issues. The paper presents interesting results, however presentation of the study needs corrections.*
**Response: Thanks, we are very much willing to do so where necessary.**

*Introduction:
Second paragraph describes bus conductors work. It does not belong to Introduction section. In this section would be expected justification of the study with presentation of hypothesis and research questions, which is missing. There is lack of presentation of the research problem, that the presented study want to solve.*

**Response: This section gave the background of the study. It will be necessary to create this background for a better appreciation of the research problem and subsequent justification of this study. The research hypothesis/question has however been provided. Thank for the observation. (see page 3, lines 22-26).**

**The research problem is contained in page 3 but more vividly stated in lines 18-22**

*Method
2.1. participants: “Thus, N = 2(1.96 +0.84)2 / (0.21)2 = 15.68 / 0.0441 = 355.56” – presenting this calculations is too trivial.*

**Response: This has been removed. Thanks for the observation.**

*“Some ergonomic concepts such as reach, clearance, awkward and constrained postures, symptoms of musculoskeletal disorders, job stress indicators etc were explained to the participants using lay terms.” – how those concepts were defined?*

**Response: These definitions have been added to the study (page 6)**

*Results
Results are presented in tables. The same time a lot of those data values are repeated in the text. It is not excessive and unnecessary?
“Prediction Model for Work Related Musculoskeletal Disorders” – why the equation (model) is not presented?*

**Response: The unnecessary repetitions have been deleted. Thanks for the observation**

**Logistic regression was done to determine the odds of developing WMSD and the model has been described and shown in table 5. This is unlike a multiple regression or a linear regression cannot be represented by a linear equation.**

*Discussion of study limitations is missing.*

**Response: Thanks, this has been added (see page 17)**

**Reviewer #3:**

*1. Indication of minibus drivers as bus drivers is not a proper comparison, these concepts should be detailed, perhaps also in the topic of the article, the differences between the work of a bus driver and a minibus are indirectly confirmed by the research cited in the article: "Contrarily, studies by Sekkay et al, (16) ) and Kärmeniemi et al, (17) reported a lower WMSD prevalence among commercial drivers in Canada and Finland respectively. "*
**Response: Thanks, this change has been made**

*2. Average parameters of the heart work indicate a healthy group of people in this respect, also taking into account the age of the respondents, which does not confirm musculoskeletal problems, especially in over 95% of employees.*
**Response: Heart rate is not a sole determinant of health (a multifaceted construct). Also younger persons are able to accommodate both physical and mental workload without a significant change in heart rate. Finally, heart rate and age were not found to be predictive of WMSD.**

*3. It is worth emphasizing in the article that most of the respondents worked 6 or more days a week, only 10 of the respondents worked 4 or 5 days a week.*

**Response: Thanks for this insight. It has been emphasized (page 7)**

Overall, we are very pleased with our revised manuscript based on the recommendations of the reviewers. We thank you for your interest and continued consideration of our application

Most respectfully,

Dr. Ekechukwu E.N.D

Department of Medical Rehabilitation, FHST,

College of Medicine, University of Nigeria.

([nelson.ekechukwu@unn.edu.ng](mailto:nelson.ekechukwu@unn.edu.ng))
