# Supplementary material for: Ergonomic assessment of work-related musculoskeletal disorder and its determinants among commercial mini bus drivers and driver assistants (mini bus conductors) in Nigeria
Source: PLoS One. 2021 Dec 7;16(12):e0260211. doi: 10.1371/journal.pone.0260211 (PMC8651118; doi:10.1371/journal.pone.0260211)
Supplement: Attachment — Submitted filename: Response to Reviewers.docx [file pone.0260211.s002.docx]

**September 29th, 2021**

**Editor, PLOS One**

Dear Editor:

Thank you for your interest in our manuscript (PONE-D-21-05678) titled, *"Ergonomic Assessment of Work-Related Musculoskeletal Disorder and its Determinants among Commercial Mini Bus Drivers and Driver Assistants (Mini Bus Conductors) in Nigeria".* We have carefully read your comments and recommendations and find them to be helpful. We have taken great care to present our commentary with accuracy, precision, and clarity.

To facilitate your evaluation of our revised manuscript, we have incorporated all the reviewers’ comments (*italicized*) in our reply **(bold)** and addressed the requests with specific point-by-point responses to each query. We separately delineated and numbered each query with a corresponding response. All editions, additions and changes in the manuscript were done using the MS Word Track change Highlight**.** We also indicated the precise location in the revised manuscript where we have addressed your comments. All references to the location of our changes (i.e., page) correspond to the revised manuscript.

1. If the authors have adequately addressed your comments raised in a previous round of review and you feel that this manuscript is now acceptable for publication, you may indicate that here to bypass the “Comments to the Author” section, enter your conflict of interest statement in the “Confidential to Editor” section, and submit your "Accept" recommendation.

*Reviewer #1: (No Response)*

*Reviewer #2: All comments have been addressed*

*Reviewer #3: All comments have been addressed*

**Response: Thank you.**

2. Is the manuscript technically sound, and do the data support the conclusions?
The manuscript must describe a technically sound piece of scientific research with data that supports the conclusions. Experiments must have been conducted rigorously, with appropriate controls, replication, and sample sizes. The conclusions must be drawn appropriately based on the data presented.

*Reviewer #1: Yes*

*Reviewer #2: Yes*

*Reviewer #3: Yes*

**Response: Thank you.**

3. Has the statistical analysis been performed appropriately and rigorously?

*Reviewer #1: Yes*

*Reviewer #2: Yes*

*Reviewer #3: Yes*

**Response: Thank you.**

4. Have the authors made all data underlying the findings in their manuscript fully available?

The [PLOS Data policy](http://www.plosone.org/static/policies.action#sharing) requires authors to make all data underlying the findings described in their manuscript fully available without restriction, with rare exception (please refer to the Data Availability Statement in the manuscript PDF file). The data should be provided as part of the manuscript or its supporting information, or deposited to a public repository. For example, in addition to summary statistics, the data points behind means, medians and variance measures should be available. If there are restrictions on publicly sharing data—e.g. participant privacy or use of data from a third party—those must be specified.

*Reviewer #1: No*

*Reviewer #2: Yes*

*Reviewer #3: No*

**Response: Thank you, a data repository link (**[**https://data.mendeley.com/drafts/brt3myjxbm**](https://data.mendeley.com/drafts/brt3myjxbm)**) as well as citation (EKECHUKWU, Echezona Nelson Dominic (2021), “WMSD among Bus Drivers and Conductors”, Mendeley Data, V1, doi: 10.17632/brt3myjxbm.1) for the data have been provided (See page 20, lines 9-12)**

5. Is the manuscript presented in an intelligible fashion and written in standard English?
PLOS ONE does not copyedit accepted manuscripts, so the language in submitted articles must be clear, correct, and unambiguous. Any typographical or grammatical errors should be corrected at revision, so please note any specific errors here.

*Reviewer #1: Yes*

*Reviewer #2: Yes*

*Reviewer #3: Yes*

**Response: Thank you.**

6. Review Comments to the Author
Please use the space provided to explain your answers to the questions above. You may also include additional comments for the author, including concerns about dual publication, research ethics, or publication ethics. (Please upload your review as an attachment if it exceeds 20,000 characters)

*Reviewer #1: 1. I suggest that the hypotheses are presented in a list to make them more visible and to achieve a clear structure.
2. Why does the hypothesis expect a higher prevalence of WMSD among bus drivers rather than bus conductors? Please explain in more detail. This seems confusing because the literature review stated that bus conductors have even worse working postures. Shouldn’t they be the ones showing higher prevalence for WMSD?
3. The second hypothesis talks about "these cohorts". Which cohorts are meant here? Bus drivers, conductors of both? Please make this more clear.*

**Response: Thank you.**

1. The hypotheses have been presented in a list as suggested (see page 3, lines 21 and 22)

2. It is only a hypothesis, it can swing in any direction. However, this has been re-stated to make it less confusing. (see page 3, lines 22 and 23)

3. The cohort here refers to bus drivers and bus conductors. This has been restated for clarity (see page 3, line 23)

*Reviewer #2: (No Response)*

**Response: None**

*Reviewer #3: I'm not sure if I understand the wording right "Commercial Mini Drivers". Please check if the change in the description of the studied group of people has been taken into account throughout the article, eg in conclusions.*

**Response: Thank you. The term “mini bus” was suggested inorder to further delineate the type of bus operated by the participants. Your comment on inconsistent use is well noted and appreciated. These have been corrected throughout the manuscript.**

7. PLOS authors have the option to publish the peer review history of their article ([what does this mean?](https://journals.plos.org/plosone/s/editorial-and-peer-review-process#loc-peer-review-history)). If published, this will include your full peer review and any attached files.
If you choose “no”, your identity will remain anonymous but your review may still be made public.
**Do you want your identity to be public for this peer review?** For information about this choice, including consent withdrawal, please see our [Privacy Policy](https://www.plos.org/privacy-policy).

*Reviewer #1: No*

*Reviewer #2: No*

*Reviewer #3: No*

**Response: None**

Overall, we are very pleased with our revised manuscript based on the recommendations of the reviewers. We thank you for your interest and continued consideration of our application

Most respectfully,

Dr. Ekechukwu E.N.D

Department of Medical Rehabilitation, FHST,

College of Medicine, University of Nigeria.

([nelson.ekechukwu@unn.edu.ng](mailto:nelson.ekechukwu@unn.edu.ng))
